# Supplementary material for: Gender- and discipline-specific bibliometric analysis of elite biomedical scientists: how is pharmacology performing?
Source: Naunyn Schmiedebergs Arch Pharmacol. 2025 Jun 12;398(12):17537–59. doi: 10.1007/s00210-025-04360-z (PMC12678612; doi:10.1007/s00210-025-04360-z)
Supplement: Supplementary file 1 — (DOCX 150 KB) [file 210_2025_4360_MOESM1_ESM.docx]

**Gender- and discipline-specific bibliometric analysis of elite biomedical scientists: how is pharmacology performing?**

Louisa Christin Fox^1^, Roland Seifert^1*^

^1^Institute of Pharmacology, Hannover Medical School, Carl-Neuberg-Str. 1, 30625 Hannover, Germany

*Corresponding author email: seifert.roland@mh-hannover.de

**Abstract**

In two recent studies (https://pubmed.ncbi.nlm.nih.gov/38652280/; https://pubmed.ncbi.nlm.nih.gov/38864907/) we bibliometrically analyzed Nobel laureates and top pharmacologists in Germany and found massive underrepresentation of women. In this follow-up study, using *www.research.com* as a source, gender relations in elite biomedical research were analyzed at a global level. A total of 9791 researchers from 29 countries from the main category of biology and biochemistry were analyzed. The pooled global data set results in a proportion of 14.9% women among the elite researchers; in the individual countries, it ranged from 31.2% (Italy) to 0% (Egypt, Iran, Nigeria; however, only very small sample sizes here). Pharmacology is characterized by lower bibliometric indices than biology and biochemistry; i.e., pharmacologists have a less prominent international perception in science than other fields. Pharmacologists must generate more output to achieve a similar status to other disciplines. The analysis using common bibliometric parameters and the newly defined h/P-Index revealed that women in biology and biochemistry globally have a significantly higher h/P-Index than men—they are characterized by a more consistent scientific quality. Women are “doing well” in bibliometric analyses, achieving a good position in science measured by bibliometric parameters, despite the strong underrepresentation in elite science. In conclusion, caution must be exerted when comparing disciplines and countries bibliometrically because they have different cultures, and women are differentially represented in elite research in various countries.

**Keywords**: Bibliometric comparison, Gender research, Gender equality, Elite research, h-Index

**Abbreviations**

B Beta-coefficient

C Citations

C/P-Index Quotient from citations by publications

D-Index Discipline Hirsch-Index

Fig. Figure

h Hirsch-Index

h-Index Hirsch-Index

h/P-Index Quotient from Hirsch-Index by publications

max Maximum

min Minimum

p *p*-value

P Publications

QQ plot Quantile-quantile plot

R&D Research and development

SD Standard deviation

SE Standard error

UNESCO United Nations Educational, Scientific and Cultural Organization

US(A) United States (of America)

VIF Variance inflation factor

**Introduction**

In science, as in other areas of social life, gender issues are becoming increasingly important. Statistics published in 2019 by the UNESCO Institute for Statistics with data from 2016 show that the proportion of women in research and development (R&D) is 29.3% globally (UNESCO Institute for Statistics 2019). However, this generalized figure does not consider various aspects, including horizontal and vertical segregation (Alonso et al. 2016, Mabandla 2001, Luukkonen-Gronow 1987). Horizontal segregation means that there are certain “masculine fields” in science, including the hard sciences—for example, physics or computer science. The reverse side shows that “feminized fields” also exist: biology, social sciences, and increasingly medicine. This leads to different women’s quotas on the horizontal level, for example, the different faculties of a university. Vertical segregation describes the decreasing proportion of women in higher career positions—the typical hierarchy. It is referred to as “leaky pipeline”: there are many women at student level, while there are fewer women in the positions above—the “supply” is therefore “lost” in the leaky pipe (Alonso et al. 2016; Boshoff 2005).

In bibliometric analyses, which deal with publications and the evaluation of scientific work, there are various parameters, which have been described in previous studies (Fox and Seifert 2024, Bünemann and Seifert 2024; also see Table S1). We have defined the h/P-Index as a new analysis parameter, which is a quotient of the h-Index and the number of total publications. The meaning of the new parameter is discussed below.

*Research.com*, founded by computer science professor Imed Bouchrika, defines itself as the “number one research portal for scientists.” The company’s mission is as follows: “Our mission is to make it easier for professors, research fellows, and those studying for a PhD or a master’s degree to progress with their research and to ensure they are always up to date with the latest conferences around the world and publications related to their work..” The popular scientist ranking on this website is based on the h-Index, which was developed by the physicist Jorge E. Hirsch (Hirsch 2005). At the time of data collection, this was declared as the h-Index on the website. In the meantime, *Research.com* has switched to the D-Index (discipline h-Index), which is a discipline-specific h-Index. There is also information on the researcher’s place of employment and the total number of citations and publications. For each entry, there is a brief overview of the scientist’s main areas of research, as well as their most cited articles.

The scientist ranking on *Research.com* was analyzed to evaluate the elite research in the field of biology and biochemistry (including pharmacology) more precisely. The main category of biology and biochemistry was chosen because a subanalysis of the detailed disciplines can be carried out—including elite pharmacology. Because of vertical segregation, a lower proportion of women was to be expected than indicated by the data from the UNESCO Institute for Statistics. The countries included in the analysis were initially determined based on the number of entries on *Research.com*, meaning countries that are highly active in research are particularly well represented. The traditional research nations can be found with large sample sizes. The selection of countries with a smaller data set is based on historical characteristics of the country and its importance in current science. Furthermore, the selection process ensured that worldwide data was collected, resulting in an overview of African, South and North American, Asian, and European research.

The following research questions will be addressed:

1. Are there country-specific differences in the proportion of women in elite science?
2. How do pharmacologists rank among elite scientists?
3. How do women perform in bibliometric analyses?
4. How comparable and fair are bibliometric analyses? Is the h/P-Index contributing useful information in bibliometric comparisons?

**Materials and methods**

A flowchart of the methodical procedure is shown in Fig. 1. The data collection was carried out by importing the data from *Research.com* into an Excel file. As the assignment of gender at *Research.com* in the information about the researchers is sometimes incorrect, the identification is based on other methods. In German-speaking and most European countries, the assignment was often intuitively very easy. The images, which were not always available, also supported the gender characterization. Problems arose particularly in the Asian countries represented in the data set, as many of the names are used gender-neutrally (Zehetbauer et al. 2022). Other images that could be found on the internet or pronouns were used to support the categorization. However, in 134 cases, no gender categorization was possible (Fig. 2): these scientists were excluded from further analysis. A further reflection of the gender assignment and its limitations can be found below. The data was checked for duplicates, which were deleted if present. The women’s share was analyzed using Excel, as were calculations of the h/P-Index and C/P-Index of the respective researcher. A table of the top scientists in a country specializing in biology and biochemistry with national rank, world rank, name, institution, h-Index, citations, publications, h/P-Index, C/P-Index, and gender was generated. The further analyzed data consists of 9657 scientists from 29 countries, of which 8217 are male and 1440 female. A more detailed overview of the data set can be found in Table S2. An additional analysis of the global top 100 should determine discipline-specific differences in elite research. Because of certain observations regarding the women’s share and respective mean values, the big data sets were analyzed in the “lower half.” A detailed explanation of this analysis can be found below.

**Fig. 1** Graphical illustration of the workflow

**Fig. 2** Overview of the analyzed data. A total of 9791 researchers from *Research.com*’s “best scientist” ranking were included in the data set. It was not possible to identify the gender of 134 people. Percentages indicated in the proportions of the pie chart

The data were analyzed in more detail using GraphPad Prism version 9/10. The original parameters were analyzed for correlations: citations (x) vs. publications (y), publications (x) vs. h-Index (y), and h-Index (x) vs. citations (y).

In addition, several parameters were tested for significance. The corresponding data were first tested for normal distribution. The D'Agostino-Pearson test was mainly used, but all results were considered (GraphPad Software n.d.). For very large amounts of data or unclear results, more emphasis was placed on visual inspection of the quantile-quantile plot or histogram of the distribution, as even small deviations from the normal distribution can cause the significance of the tests to fail (Ghasemi and Zahediasl 2012). Depending on the results of the visual inspection of the QQ plot and the results of the normal distribution tests, the Mann-Whitney test was used for non-normally distributed data, and the unpaired *t*-test with Welch’s correction, if necessary, for normally distributed data. The individual countries as well as the pooled continental and global data sets were analyzed, and finally multi-linear regressions were performed for the global top 100 and the Japanese and French data sets to examine correlations between variables. Significant results were then tested for robustness by excluding the top 1% of each dataset. All results were constant except for one result in Table 12 (see below).

**Results and Discussion**

*Women in elite science in international comparison*

Figure 3 shows the women’s quotas among the analyzed scientists and Fig. 4 the total numbers. There are three broad categories: the top with more than or equal to 20% women, the bottom with less than or equal to 10%, and the mid-range in between. The top countries roughly have a European focus, while the bottom countries tend to have an Asian focus.

Seventy-five percent of the top 100 scientists worldwide come from the USA (see Fig. S1). Research in the USA is at a very high level. The US Top 1000 ends with an h-Index of 80, whereas in other countries, this value would rank first in the list. This explains the high proportion of US scientists in the Top 100. The 5 women in the top 100 are all employed in the USA. This results in a female quota of 5% among the 100 “best scientists” on *Research.com*. Another aspect of US-American science is revealed: the USA serves as a promising country for international researchers (Grigat 2018), resulting in only one woman of the global top five women also being a native US-American.

**Fig. 3** Overview of the women’s share in the countries included in the analysis. Sorted from highest (left; 31.2% in Italy) to lowest women’s share (right; Iran, Egypt, and Nigeria with 0% each). The percentages and total numbers are given excluding the scientists who could not be assigned a gender, resulting in a total female share of 14.9%

**Fig. 4** Overview of the absolute numbers of analyzed scientists in the countries. Sorted from the highest number of male researchers (left; 919 males in Japan) to the lowest number of male researchers (right; 1 male in Nigeria). The sample sizes are indicated above the bars

*How elite pharmacologists can compete with other disciplines*

Disciplines that could be assigned to the top 100 scientists of the cohort are shown in Fig. 5. As it was often not possible to assign them to a specific field, multiple mentions occur. The biology cluster is best represented (21.56%), as are neurosciences (11.38%) and biochemistry and molecular sciences (10.78% each).

**Fig. 5** Visualization of the different disciplines of the global top 100 of the “best scientist” ranking on *Research.com* in the main category biology and biochemistry. There are over 100 mentions of disciplines in the graph because the scientists were often not assigned to one but to several disciplines

The field of pharmacology and toxicology is represented with 4.19% (7 mentions). In an analysis of the non-peer-reviewed magazine *Biospektrum* (Zöllner and Seifert 2023), around 2.4% of publications could be assigned to pharmacology. Thus, pharmacology represents a rather small field within the analyzed data. The present analysis emphasizes that the international perception of pharmacologists is different from that of more popular disciplines. Pharmacologists are not as well represented in the global top 100 list as more popular research fields, as the perception in the different scientific subfields is different. Of these 7 mentions of pharmacology and toxicology, all researchers are male, resulting in a proportion of 100% male pharmacologists. In a recent study, we analyzed the elite members of the German Society for Experimental and Clinical Pharmacology and Toxicology (DGPT), who are represented in the German “best scientist” ranking in biology and biochemistry on *Research.com*. 4.2% of the German elite researchers in biology and biochemistry are members of the DGPT (Fox and Seifert 2024). Once again, the small size of the pharmacological discipline is underlined. Table 1 compares these aspects of the top 100/pharmacology subanalysis with the studies by Zehetbauer et al. (2022), Zöllner and Seifert (2023), and Fox and Seifert (2024).

**Table 1** Comparison of the pharmacology subanalysis with the studies by Zehetbauer et al. (2022), Zöllner and Seifert (2023), and Fox and Seifert (2024). The studies by Zehetbauer et al. and Zöllner and Seifert examined the publication behavior in the journals *Naunyn-Schmiedeberg’s Archives of Pharmacology* (peer-reviewed) and *Biospektrum* (non-peer-reviewed) over a certain period and established year-specific, discipline-specific, and geographical correlations. As the previous analysis of the elite members of the DGPT (Fox and Seifert 2024) and the present analysis cover both historically and presently significant research, are less year-specific than the other studies, and cover elite research, the proportion of women is comparatively low

| Aspect | Zehetbauer et al. (2022) | Zöllner and Seifert (2023) | Fox and Seifert (2024) | Present study—subanalysis |
| --- | --- | --- | --- | --- |
| Data source | *Naunyn-Schmiedeberg’s Archives of Pharmacology* | *Biospektrum* | *Research.com*’s “best scientist” ranking—elite members of the DGPT | *Research.com*’s “best scientist” ranking—global Top 100 |
| Years analyzed | 2000–2020 | 1999–2021 | Data collection in 2022 | Data collection in 2022 |
| Country | Germany | Germany | Germany | 12 countries |
| Specializations | 1 (Pharmacology) | 29 | 1 (Pharmacology) | 21 |
| Persons analyzed and results | 2886 authors  - 2071 male (71.8%)  - 815 female (28.2%)  Including 651 first authors  - 443 male (68.0%)  - 208 female (32.0%)  And 651 senior authors  - 574 male (88.2%)  - 77 female (11.8%) | 3197 authors  - 2147 male (67.2%)  - 1050 female (32.8%)  Including 76 pharmacologists  - 62 male (81.6%)  - 14 female (18.4%) | 42 members of the DGPT in the German ranking (4.2% of biologists/biochemists)  - 40 male (95.2%)  - 2 female (4.8%) | 100 researchers  - 95 male (95%)  - 5 female (5%)  Including 7 pharmacologists  - 7 male (100%)  - 0 female (0%) |

In a further approach, we compared the mean values for h-Index, total citations, and total publications, as well as the age of the scientists in 2022 for the different specializations. Firstly, tables were created with the scientists who can be assigned to the respective specialization. All specializations with more than five researchers were included (see Fig. 5). The age of the scientists was determined using information from CVs or short biographies from the internet. This was not possible for all the researchers. As the data collection took place in 2022, the age of researchers who passed away before was extrapolated to 2022, as the h-Index can also increase after a scientist’s death. Individual statistical tests were carried out to compare the mean values of the primary bibliometric parameters and the age. Specific focus is given to pharmacology: can the small discipline measure up against the larger disciplines of this analysis? Figure 6a–d and Table 2 show the results of the analyses. After testing for normal distribution, either an unpaired *t*-test (with Welch’s correction if needed) or a Mann-Whitney test was performed. For the analyses with the comparative cohort “All specializations,” the Kruskal-Wallis test was performed due to the lack of normal distribution. To determine the statistical parameters of the “All specializations” group, the multiple mentions of scientists who can be assigned to several specializations were only counted once. If the “All specializations” group represents a comparative cohort, there is no significance between the individual disciplines and the main category in any bibliometric parameter or age. Only the citations (Fig. 6b) are significantly higher for oncologists than for biologists, neuroscientists, biochemists, molecular scientists, and pharmacologists. The oncologists are also significantly younger than the neuroscientists, biochemists, and pharmacologists, while the biologists are significantly younger than the biochemists. These analyses indicate that pharmacology can establish itself as a small discipline in the large main category of biologists and biochemists in the ranking. The h-Index and the citations are in the lower range. It is particularly apparent that the number of publications—although insignificant—s well below that of the other disciplines, and the average age of pharmacologists is also the highest. This allows the assumption that pharmacological and toxicological publications represent more effort for the same outcome. In any case, it underlines the different importance of bibliometric parameters in science. Sometimes publications are not comparable because they have a different value; the same can apply to citations. Comparisons between several disciplines reveal subject-specific differences in bibliometrics.

**Fig. 6 a–d** Comparison of the bibliometric parameters h-Index (**a**), citations (**b**), and publications (**c**), as well as the age of the scientists in 2022 (**d**) between the most represented disciplines in the global top 100 in the “best scientist” ranking on *Research.com* in the top category of biology and biochemistry. Indicated above the bars are the means of the analyzed group; the whiskers show the standard deviation. Only significant differences are marked. Legend: * = *p*≤0.05; ** = *p*≤0.01

**Table 2** Statistical analysis of the comparison of disciplines between the most represented specializations of the global top 100 of the “best scientist” ranking on *Research.com* in the top category biology and biochemistry. Please note that the number of scientists N in the group “All specializations” is lower than the number of groups above combined due to multiple mentions of disciplines for an individual scientist

| Specialization (*N*) | h-Index mean (SD) | Citations mean (SD) | Publications mean (SD) | Age (SD) |
| --- | --- | --- | --- | --- |
| Biology (36) | 164.55 (22.50) | 131,395.06 (57,535.76) | 574.84 (250.03) | 70.48 (10.61) |
| Neurosciences (19) | 168.50 (23.77) | 110,026.94 (35,680.33) | 523.67 (181.01) | 76.50 (9.09) |
| Biochemistry (18) | 173.22 (31.80) | 123,100.22 (44,528.97) | 552.06 (179.51) | 77.56 (9.08) |
| Molecular sciences (18) | 165.94 (33.46) | 133,766.33 (71,591.88) | 549.33 (288.21) | 71.38 (10.04) |
| Oncology (16) | 172.75 (21.64) | 177,866.56 (67,545.22) | 513.81 (178.66) | 67.18 (10.47) |
| Immunology (10) | 177.80 (43.58) | 159,562.60 (101,428.54) | 612.30 (295.95) | 72.57 (10.13) |
| Pharmacology (7) | 164.86 (14.96) | 122,568.71 (53,205.46) | 448.71 (93.66) | 78.86 (9.35) |
| Genetics (7) | 173.14 (25.78) | 143,499.57 (34,979.73) | 522.14 (174.74) | 72.33 (9.18) |
| Pathology (6) | 172.00 (26.26) | 175,266.33 (72,343.70) | 556.50 (233.71) | 74.75 (6.95) |
| All specializations (94) | 169.51 (27.21) | 136,112.77 (61,574.44) | 549.40 (223.79) | 72.21 (9.80) |

*What are the characteristics of a Nobel laureate?*

Table 3 compares the male with the female top 5 of the “best scientist” ranking on *Research.com* globally vs. the Nobel laureates analyzed by Bünemann and Seifert (2024) in a recent study. A detailed overview of the male and female top five of the ranking can be found in Tables S3 and S4. The collected data is suitable for a comparison even though the analyzed cohort is different: both studies highlight extraordinary science, but the *Research.com* ranking, for example, does not include any female Nobel laureates and only one male Nobel laureate, and the ranking by h-Index or the rating of a scientist with the h-Index is to be considered independently of the Nobel Prize question (Bünemann and Seifert 2024). There is one Nobel laureate in the data of the present analysis, Robert J. Lefkowitz, who was awarded the Nobel Prize in chemistry in 2012 together with Brian Kobialka for his discoveries on G protein-coupled receptors. The study by Bünemann and Seifert represents a snapshot of when a prize was awarded, while the analysis of the *Research.com* ranking describes a more dynamic context.

Firstly, the parallels between the ten more detailed analyzed scientists of the present study will be disclosed. Two scientists conduct joint research with their spouses, who are also recognized as elite scientists on *Research.com*. Three scientists are of Jewish origin, among them two women who were directly affected by the Holocaust. The Nobel laureate Robert Lefkowitz joins the ranks of the numerous Nobel laureates of Jewish origin. In the period 1901 to 2023, 26% of Nobel Prize winners in the field of medicine and physiology and 19% in the field of chemistry were of Jewish origin. This percentage is even more meaningful considering that only 0.2% of the world’s population is Jewish (VTC-Nachrichten 2023). No pharmacologists appear in the top five females and males; the most represented disciplines are the biology field, the neurosciences, and biochemistry (three mentions each; 18.75%).

The average age of the top five men is 66.6, while the average age of the top five women is 78. All the scientists in the male list are still alive, while two women have already passed away. The age of the two deceased scientists was extrapolated to the year 2022 to calculate the mean value for the group. That the top five women in the list are on average almost 12 years older than the top five men in the ranking contrasts with the statement of the study by Bünemann and Seifert (2024), which deals with the Nobel laureates in the fields of medicine and physiology as well as pharmacologically relevant topics of Nobel laureates in chemistry. The female laureates were on average more than 7 years younger at the time of the ceremony (60.1 years female, 67.4 years male). However, the restriction from above applies again: the analyzed cohorts differ in their composition and meaning.

All three bibliometric parameters are higher for the men in the *Research.com* ranking. This is self-explanatory given that the first woman in the global top 100 list only appears in 47th place. The average h-Indices of the *Research.com* scientists are higher than those of the Nobel laureates. Some of the scientists analyzed by Bünemann and Seifert (2024) had an h-Index of 0—the significantly higher values in the *Research.com* list are explained by the basis of the ranking and it explicitly relies on the highest h-Indices, while the awarding of a Nobel Prize is not based on bibliometrics. It shows that one does not necessarily have to have an extraordinary h-Index to be awarded a Nobel Prize for groundbreaking scientific discoveries. *Research.com* scientists have significantly higher values for the mean publications. This shows that the scientific quantitative output is also independent of the Nobel Prize.

In terms of origin and the location of employment, the USA is very well represented. In all subgroups, there are more scientists working in the USA than there are US natives. The origin of the scientists is more diverse. This emphasizes the point made above: the USA is a research stronghold and is suitable for emigration for professional reasons (Grigat 2018). Other countries of employment represented are the typical research nations, all of which were covered by this dataset.

The disciplines to which the Nobel laureates belong were further analyzed as shown in Fig. 5 for the top 100 of the *Research.com* ranking. The results of this discipline analysis are shown in Fig. 7. The general disciplines represented are roughly the same. There are fewer neuroscientists and oncologists among the Nobel laureates, more chemists, microbiologists, and geneticists, as well as a few more clinical subjects (as the Bünemann and Seifert data set also includes Nobel laureates in physiology or medicine). There is only one (female) pharmacologist among the Nobel laureates.

**Table 3** Comparison of the most important aspects of the top five men vs. top five women vs. Nobel laureates analyzed by Bünemann and Seifert (2024). The mean publications for Bünemann and Seifert (2024) are the added values of mean publications before and after the awarding of the Nobel Prize

| Aspect | Present study (*Research.com*) | | Bünemann and Seifert (2024) (Nobel laureates) | |
| --- | --- | --- | --- | --- |
|  | *Top 5 male* | *Top 5 female* | *Male (N=45)* | *Female (N=10)* |
| Mean age in years (min–max) | 66.6 (46–84) | 78 (75–80) | 67.4 (46–85) | 60.1 (48–84) |
| Mean h-Index (min–max) | 245 (226–281) | 152.8 (145–163) | 90.2 | 78.8 |
| Mean citations (min–max) | 242,601 (200,537–398,396) | 96,741.2 (77,974–149,535) | / | / |
| Mean publications (min–max) | 918.4 (763–1380) | 551.2 (299–848) | 333.3 | 321.5 |
| Origin | 40% Germany  40% USA  20% New Zealand | 20% Taiwan  20% Germany  20% Czech Republic  20% Israel  20% USA | 51.1% USA  15.6% UK  8.9% Japan  4.4% Germany  2.2% each France, Sweden, Norway, Denmark, Canada, India, Italy, Ireland, Luxembourg | 40% USA  20% France  10% Israel  10% China  10% Australia  10% Norway |
| Country | 60 % USA  20 % France  20 % Germany | 100 % USA | 57.8% USA  17.8% UK  13.3% Japan  4.4% Germany  2.2% each Canada, Norway, Denmark | 50% USA  10% France  10% Israel  10% China  10% Germany  10% Norway |

**Fig. 7** Visualization of the different disciplines of the Nobel laureates analyzed by Bünemann and Seifert (2024). There are over 55 mentions of disciplines in the graph because the scientists were often not assigned to one but to several disciplines

*How do elite female scientists perform in bibliometric analyses?*

The average h-Index for women is higher than for men in only three countries: Finland, Norway, and Turkey (here, however, the female group consists of only one person). Women are often found in the “lower” h-Index areas and often do not top the list; therefore, the mean h-Index is usually lower for women. On average, only women from Finland, Hungary, South Korea, Norway, and Turkey have higher citations than men. In terms of publications, women from Denmark, Finland, Norway, and Russia have higher mean values than men. The C/P-Index, like the h/P-Index, is on average higher for many women than for men. For the C/P-Index, this applies to Australia, Belgium, Canada, China, Germany, Spain, Finland, France, Hungary, Israel, Italy, South Korea, the Netherlands, Norway, Sweden, Turkey, and the USA. For the h/P-Index, the mean value is only higher for men in Austria, Denmark, Hungary, Norway, and Russia. For a detailed overview of the bibliometric parameters, see Table S5.

The data from *Research.com* was transferred to the Excel spreadsheets in steps of 100. The proportion of women was then analyzed for these intervals and evaluated in the ranking from top to bottom. Countries in which the share of women fluctuates evenly around a mean value and is not considerably lower in the upper ranks and increases slightly towards the bottom are characterized by smaller differences between the male vs. female bibliometric parameters. Examples of this are Spain, Finland, Norway, and Poland. The hypothesis is that a further distinction must be made between very top and “lower” top in the elite of researchers. As the top ranks in the *Research.com* ranking are mainly occupied by men due to various factors discussed below, these are to be regarded as outliers in the summarized analysis in a generalized context, which strongly distorts the picture. The analysis of the “lower half” of the elite group is supposed to find out how women are establishing themselves at the top of research and how their performance in bibliometric analyses compares to men.

For this analysis, the large data sets with over 500 entries on *Research.com* (Australia, Canada, Germany, France, Japan, UK, USA) were split in half and the “lower half” was statistically analyzed in a new document. The composition of these data sets is shown in Table 4. The results of the statistical analysis of the primary bibliometric parameters (using either the unpaired *t*-test with Welch’s correction if needed for normal distributed or the Mann-Whitney test for non-normally distributed data) are shown in Tables 5 (h-Index), 6 (citations), and 7 (publications).

**Table 4** Absolute and relative composition of the data sets for the bibliometric analysis of the “lower half”

| Country | *N* male (%) | *N* female (%) | *N* total |
| --- | --- | --- | --- |
| Australia | 203 (76.9%) | 61 (23.1%) | 264 |
| Canada | 247 (82.1%) | 54 (17.9%) | 301 |
| Germany | 426 (85.4%) | 73 (14.6%) | 499 |
| France | 311 (71.8%) | 122 (28.2%) | 433 |
| Japan | 454 (94.8%) | 25 (5.2%) | 479 |
| UK | 414 (83.1%) | 84 (16.8%) | 498 |
| USA | 446 (89.2%) | 54 (10.8%) | 500 |

**Table 5** Statistical analysis of the h-Index for the countries with more than 500 entries for the “lower half” of the top scientists

| Country | Mean h-Index male (SD) | Mean h-Index female (SD) | *p*-value |
| --- | --- | --- | --- |
| Australia | 44.55 (3.584) | 45.02 (3.580) | 0.3005 |
| Canada | 45.10 (3.981) | 45.83 (3.549) | 0.1435 |
| Germany | 50.39 (3.905) | 50.26 (3.633) | 0.8207 |
| France | 43.85 (2.908) | 44.08 (3.022) | 0.5036 |
| Japan | 49.51 (3.585) | 49.52 (3.721) | 0.9979 |
| UK | 51.57 (3.304) | 51.13 (3.407) | 0.2571 |
| USA | 87.46 (4.681) | 86.04 (4.472) | 0.0313 (*) |

**Table 6** Statistical analysis of the citations for the countries with more than 500 entries for the “lower half” of the top scientists

| Country | Mean citations male (SD) | Mean citations female (SD) | *p*-value |
| --- | --- | --- | --- |
| Australia | 9170 (4350) | 9738 (5196) | 0.4427 |
| Canada | 9290 (4112) | 10,652 (8859) | 0.1580 |
| Germany | 10,725 (5142) | 12,106 (6612) | 0.1330 |
| France | 8531 (3652) | 9261 (5053) | 0.6187 |
| Japan | 10,198 (4831) | 9350 (3627) | 0.2024 |
| UK | 11,707 (6526) | 12,022 (5345) | 0.0834 |
| USA | 32,736 (16,632) | 34,339 (13301) | 0.5811 |

**Table 7** Statistical analysis of the publications for the countries with more than 500 entries for the “lower half” of the top scientists

| Country | Mean publications male (SD) | Mean publications female (SD) | *p*-value |
| --- | --- | --- | --- |
| Australia | 123.00 (41.54) | 114.10 (36.13) | 0.1444 |
| Canada | 111.90 (31.34) | 105.60 (29.62) | 0.1998 |
| Germany | 120.80 (36.76) | 108.40 (31.21) | 0.0070 (**) |
| France | 101.40 (28.22) | 99.16 (25.69) | 0.7237 |
| Japan | 144.20 (49.50) | 119.00 (34.22) | 0.0131 (*) |
| UK | 116.40 (32.33) | 110.50 (27.82) | 0.1749 |
| USA | 233.00 (73.64) | 207.40 (52.48) | 0.0166 (*) |

In comparison to Fig. 3, Table 4 shows that the share of women in the countries analyzed increases towards the bottom because the share of women in Table 4 is higher than in Fig. 3 for all countries. This supports the hypothesis that a generalized bibliometric analysis of all ranks distorts the results. However, the share of women is still lower than the share of men in all countries in Table 4. The differences observed for the h-Index in Table 5 are marginal; only in the USA is the h-Index slightly significantly higher for men (by 1.42 points). There is no significance regarding the citations (Table 6). However, the mean citations are often higher for women than for men. On average, women publish less than men in all countries, with significance in Germany, Japan, and the USA (Table 7). These constellations would lead to higher C/P- and h/P-Indices for women. This important analysis shows that women can certainly establish themselves in elite science, even if they are represented in smaller numbers.

The C/P-Index: a measure of scientific recognition?

The further bibliometric analysis is shown for the French and Japanese data comparing a country with a high women’s share and a country with a lower one. Figure 8 shows the distribution of the C/P-Index. The C/P-Index (quotient of total citations by total publications), as described by Dashun and Barabási (2021), indicates how many citations a scientist’s publications receive on average. It provides a good initial orientation. However, it does not require any correction mechanisms and can be strongly distorted by a small number of very successful publications (meaning high citation counts). This must be considered in its interpretation.

A Mann-Whitney test was performed to test whether the C/P-Index differs between the sexes, as the data does not show a normal distribution. In both countries, there is no significance in the distribution of the C/P-Index (France *p* = 0.2623; Japan *p* = 0.9106). Significance in the distribution of the C/P-Index is found in the USA, Italy, and Finland—where the C/P-Index is slightly significantly higher for women—while it is slightly significantly higher for men in Denmark (Table 8).

**Fig. 8** C/P-Index male vs. female for France (**a**) and Japan (**b**)

**Table 8** Summary of the means of the C/P-Index for male and female scientists with standard deviation in brackets and *p*-value. Only the countries with significant differences are listed

| Country | Mean male (SD) | Mean female (SD) | *p*-value |
| --- | --- | --- | --- |
| Denmark | 99.22 (58.25) | 78.71 (61.52) | 0.0019 (**) |
| Finland | 92.80 (48.91) | 119.0 (82.35) | 0.0494 (*) |
| Italy | 85.76 (61.16) | 91.56 (53.77) | 0.0249 (*) |
| USA | 176.7 (108.2) | 192.8 (93.51) | 0.0314 (*) |

*A new measure of consistency of scientific productivity*

The h/P-Index is shown in Fig. 9 for the French, Japanese, and pooled global data. The h/P-Index is the quotient of h-Index by total publications and indicates how many publications of a researcher achieve such high citations that they can contribute to the h-Index. A relative indication is given: how many publications achieve *h* citations? A Mann-Whitney test was performed to test whether the h/P-Index differs between the sexes, as the data do not show a normal distribution. In all three samples, the h/P-Index is significantly higher for females (France *p* = 0.0020; Japan *p* = 0.0025; pooled global data *p*<0.0001). In all data, women show a higher consistency in their scientific work. This should be considered against the background of the low proportion of women. In Japan, too, the h/P-Index is significantly higher for women, with a proportion of women of just 4.1%. The h/P-Index is also significantly higher for women in Australia, Canada, Germany, Spain, Italy, Sweden, the USA, and the pooled Asian and European data. For men, it is significantly higher in Denmark (Table 9).

**Fig. 9** h/P-Index male vs. female for France (**a**), Japan (**b**), and the pooled global data (**c**)

**Table 9** Summary of the means of the h/P-Index for male and female scientists with standard deviation in brackets and *p*-value. Note: only the countries with significant differences are listed

| Country | Mean male (SD) | Mean female (SD) | *p*-value |
| --- | --- | --- | --- |
| Australia | 0.3730 (0.1076) | 0.4165 (0.09851) | 0.0002 (***) |
| Canada | 0.4200 (0.1152) | 0.4604 (0.1117) | 0.0009 (***) |
| Denmark | 0.4191 (0.1236) | 0.3637 (0.1058) | 0.0327 (*) |
| France | 0.4488 (0.1140) | 0.4774 (0.1109) | 0.0015 (**) |
| Germany | 0.4236 (0.1247) | 0.4634 (0.1294) | 0.0023 (**) |
| Italy | 0.3602 (0.1080) | 0.3985 (0.1107) | 0.0008 (***) |
| Japan | 0.3618 (0.1127) | 0.4337 (0.1527) | 0.0025 (**) |
| Spain | 0.3916 (0.1091) | 0.4297 (0.1088) | 0.0024 (**) |
| Sweden | 0.4185 (0.1171) | 0.4705 (0.1135) | 0.0068 (**) |
| USA | 0.3894 (0.1112) | 0.4201 (0.1027) | 0.0051 (**) |
| Global | 0.4025 (0.1212) | 0.4341 (0.1179) | <0.0001 (****) |

Figure 10 is supposed to support these findings. For the French data, the h/P-Index interval is plotted on the x-axis against the percentage gender distribution in the interval on the y-axis. The bottom line is the unequal distribution between the sexes, as described above, but the proportion of women tends to increase in the higher h/P-Index intervals. The sample sizes of the intervals in the very low and very high ranges often consist of only a few or individual persons. However, the increase in the proportion of women in the intervals between 0.4 and 0.7 is excluded from this. This is consistent with the significantly higher h/P-Index for women in France.

**Fig. 10** h-Index-intervals (x) plotted against the percentage of genders within the intervals (y) and correlated with a quadratic regression for France

Figure 11 is also intended to support a similar statement. The publications are plotted on the x-axis against the h-Index on the y-axis. Those with a high h/P-Index are more likely to be found in the area towards the y-axis. Researchers in this area “need” relatively few publications to achieve a high h-Index, meaning that they receive a stable perception measured by constant citation figures for their publications. This correlation can be described for the French data: the female curve has a steeper rise and is found in areas associated with a higher h/P-Index. This tends to support the findings of the h/P-Index analysis: women are more likely to have a higher h/P-Index than men.

**Fig. 11** h-Index (x) plotted against publications (y) and correlated with a non-linear regression for France

In Figure 12, the h-Index on the x-axis is plotted against the citations on the y-axis and correlated for the Japanese data. The earlier rise in the female curve, which is shown particularly from an h-Index of approximately 100, can be interpreted in various ways: women are cited more overall than men, as they have more total citations with the same h-Index (I); women have more “scientific one hit wonders,” i.e., singular, highly cited successful publications, which do not drive up the h-Index but rather only the total number of citations (II); and women have a consistently high number of citations, which they receive for many of their publications, so that they have a higher scientific consistency—analogous to the h/P-Index (III). The interpretation varies from country to country. Since Japan is a country with a significantly higher h/P-Index for women, interpretation (III) is probably the closest.

**Fig. 12** h-Index (x) plotted against citations (y) correlated with a non-linear regression for Japan

*What affects the h-Index the most?*

In another approach, a multiple linear regression was performed to examine the influence of total citations, total publications, age, gender (male and female), and country of employment (USA vs. other countries) on the h-Index of the Global Top 100 of *Research.com*’s ranking of the best scientists in biology and biochemistry. The h-Index served as the dependent variable, while the independent variables were coded metrically (citations, publications, age) or categorically (gender, female = 1; country, USA = 1). Note that age could not be determined for all researchers listed. The examination of the effect of the specializations of the researchers was hindered by the inability to assign the researchers to one main discipline in some cases. The results are listed in Table 10.

The regression was overall significant (F(5, 78) = 47.38, *p*<0.0001) and explained 75.23% of the variance of the h-Index (*R²* = 0.7523). Significant variables were the number of citations (*p*<0.0001), number of publications (*p* = 0.0002), and age (*p*<0.0001). Gender (*p* = 0.1636) and country (*p* = 0.1894) were not significant.

The residuals of the regression largely met the assumption of normal distribution, as confirmed by the Anderson-Darling, Shapiro-Wilk, and Kolmogorov-Smirnov normality tests (all *p*>0.05). Only the D'Agostino-Pearson test showed a deviation (*p* = 0.0194). After inspection of the QQ plot, the normal distribution was mainly assumed for the large cohort. Multicollinearity was not a problem as all variables were found to have a variance inflation factor (VIF) between 1 and 2.

Significant variables for the scientific success of the elite researchers in the sample, as measured by the h-Index, are therefore in particular the number of citations, the number of publications, and age. Gender and country of practice had less influence, although the overrepresentation of male (95%) and US researchers (75%) in the sample should be considered when interpreting the results.

**Table 10** Multi-linear regression results for the global top 100 scientists in biology and biochemistry on *Research.com*

| Parameter estimates | Variable | Beta coefficient (B) | SE | *p* | VIF |
| --- | --- | --- | --- | --- | --- |
| β0 | Intercept | 44.31 | 14.68 | 0.0034 |  |
| β1 | Citations | 0.0003802 | 0.00003118 | <0.0001 (****) | 1.312 |
| β2 | Publications | 0.03067 | 0.007698 | 0.0002 (***) | 1.217 |
| β3 | Age | 0.7638 | 0.1716 | <0.0001 (****) | 1.144 |
| β4 | Gender [1 = female] | −9.710 | 6.904 | 0.1636 | 1.071 |
| β5 | Country [1 = USA] | 5.092 | 3.846 | 0.1894 | 1.077 |

In addition to the table, the beta coefficients of the independent variables were visualized as a bar chart (see Fig. 13). The height of the bars corresponds to the beta coefficient and the error bars correspond to the standard deviation. For better visualization, Citations × 10^−4^ and Publications × 10^−2^ were used. If a researcher’s number of citations increases by 10,000, the h-Index increases by 3.8 points on average. With 100 more publications, the h-Index increases by about 3 points. If the number of years of life increases by 1, the h-Index increases by a total of 0.76 points. On average, women have a lower h-Index (9.7 points), but this observation is not significant and must be seen in the context of the low proportion of women in the data set. Similarly, researchers from the USA have an average h-Index that is 5.1 points higher, but this correlation is also not significant.

**Fig. 13** Illustration of the beta coefficients of the investigated variables on the h-Index. For better visualization, the citation numbers were divided by 10^4^ and the publications by 10^2^. The categorical variables gender and country refer to “female” and “USA.” The heights of the bars indicate the effect size (B), while the error bars indicate the SE

Partial regression plots were created to visualize the effect of total citations, total publications, and age on the researchers’ h-Index. For this purpose, the residuals of the dependent and independent variables were first calculated, in each case excluding the residuals of the other variables. The resulting regression line shows a significant positive correlation in all three cases (see Fig. 14a–c).

**Fig. 14 a–c** Partial regression plots of the effects of numerical variables citations (**a**), publications (**b**), and researcher’s age (**c**) on the h-Index. On the x-axis, the corrected residuals of the variables are plotted and on the y-axis, the corrected residuals of the h-Index. The slope of the resulting regression line indicates the beta coefficient

The analysis was further conducted on the country datasets of Japan and France. Information regarding the h-Index, citations, publications, and gender is available for these datasets. As previously delineated, the multilinear modeling was implemented to align with the respective datasets. The graphical and tabular results are displayed in Figs. 15 and 16 and Tables 11 and 12, respectively.

**Table 11** Multi-linear regression results for the Japanese best scientists in biology and biochemistry on *Research.com*

| Parameter estimates | Variable | Beta coefficient (B) | SE | *p* | VIF |
| --- | --- | --- | --- | --- | --- |
| β0 | Intercept | 35.24 | 0.5922 | 0.0034 |  |
| β1 | Citations | 0.0007362 | 0.00002037 | <0.0001 (****) | 1.217 |
| β2 | Publications | 0.07126 | 0.003061 | <0.0001 (****) | 1.219 |
| β3 | Gender [1 = female] | 0.7047 | 1.283 | 0.5829 | 1.008 |

**Fig. 15** Illustration of the beta coefficients of the investigated variables on the h-Index of Japanese elite scientists. For better visualization, the citation numbers were divided by 10^4^ and the publications by 10^2^. The categorical variable gender refers to “male.” The heights of the bars indicate the effect size (B), while the error bars indicate the SE

The regression for the Japanese data was overall significant (F(3, 954) = 1036, *p*<0.0001) and explained 76.51% of the variance of the h-Index (*R²* = 0.7651). Significant variables were number of citations (*p*<0.0001) and the number of publications (*p*<0.0001); the gender (*p* = 0.5829) was not significant. The regression for the French data was overall significant (F(3, 862) = 828.9, *p*<0.0001) and explained 74.26% of the variance of the h-Index (*R²* = 0.7426). Significant variables were number of citations (*p*<0.0001), the number of publications (*p*<0.0001), and the gender (*p* = 0.0325).

Researchers in Japan have documented an average increase of approximately 7.4 h-Index points for every 10,000 additional citations, while in France, the increase is approximately 3.8 h-Index points for a similar number of citations. For an additional 100 publications, Japanese scientists experience an average gain of 7.1 h-Index points, while French scientists demonstrate an average increase of 11.5 h-Index points. The correlation with gender varies across countries: female French elite researchers exhibit an average decline of 1.36 h-Index points, while their female Japanese scientists demonstrate an increase of 0.7 h-Index points. The impact of gender on the h-Index of elite French researchers was only barely significant after excluding the top 1% of the data set (*p* = 0.0757).

**Table 12** Multi-linear regression results for the French best scientists in biology and biochemistry on *Research.com*

| Parameter estimates | Variable | Beta coefficient (B) | SE | *p* | VIF |
| --- | --- | --- | --- | --- | --- |
| β0 | Intercept | 34.07 | 0.6334 | <0.0001 (****) |  |
| β1 | Citations | 0.0003815 | 0.00002398 | <0.0001 (****) | 2.147 |
| β2 | Publications | 0.1150 | 0.005667 | <0.0001 (****) | 2.183 |
| β3 | Gender [1 = female] | −1.357 | 0.6336 | 0.0325 (*) | 1.025 |

**Fig. 16** Illustration of the beta coefficients of the investigated variables on the h-Index of the French elite scientists. For better visualization, the citation numbers were divided by 10^4^ and the publications by 10^2^. The categorical variable gender refers to “male.” The heights of the bars indicate the effect size (B), while the error bars indicate the SE

The results of the previously mentioned tests for normal distribution were negative for these data sets at the 0.05 level of significance. Nevertheless, the data sets are voluminous. In the context of large sample sizes, the normal distribution tests exhibit a high degree of sensitivity to minor deviations (Ghasemi and Zahediasl 2012). The data underwent logarithmic transformation in the initial phase of analysis. This transformation also revealed non-positive normal distribution tests. Following a thorough visual examination of the QQ plot and the predominantly bell-shaped distribution of the residuals of the h-Index in both data sets, the validity of the applied tests can be adequately assessed.

*The comparability of bibliometric analyses*

*Research.com* is an internet service that offers the “best scientist” ranking, along with other services. The portal is intended to provide independent information about science. Users can obtain information about the listed scientists, which can lead to potential decisions. The influence of this type of ranking can have various consequences: collaboration in science, career options like job appointments, or even funding (Ioannidis and Maniadis 2024). Because of the selected parameter, the h-Index, the data set includes historically and currently significant research. This provides a unique and broad overview of elite and meaningful research.

The underlying problem in bibliometric analysis is that there is no consensus on a suitable parameter. There is not “the one,” generally accepted way. The premise of the following study is therefore that academic performance can be assessed to some extent based on citations. The first parameter on which this analysis is based is the h-Index. This parameter determines which scientists are included in the ranking and thus in the present study. One limitation of the analyzed data set is therefore that it is based on a bibliometric parameter, which is also being discussed due to its limitations (Fox and Seifert 2024). One possible result is that some disciplines that can be assigned to the main field of biology and biochemistry are considerably more represented than smaller, less popular disciplines. It is a fact that such publications from popular fields can potentially attract more attention. This means that researchers from these disciplines—in addition to more competition—have a larger audience for the perception of their research results. There are few disciplines with many representatives in the top 100 and vice versa (Fig. 5). An analysis by Van Noorden et al. (2014) revealed that the 100 most cited papers in history often come from the field of protein biochemistry. Biochemistry and other related disciplines are also well represented in the *Research.com* Top 100 “best scientists.”

The analyses and correlations carried out with GraphPad show that there are close links between the bibliometric parameters. On the other hand, it is also clear that not every correlation can be described universally. Depending on which parameter is used for the evaluation, one researcher or the other is higher or lower on the list. In a recent study, we examined the elite members of the German Society for Experimental and Clinical Pharmacology and Toxicology (DGPT), which were represented in the German ranking of the “best scientists” in biology and biochemistry on *Research.com*. In this cohort, the scientists were ranked according to the bibliometric parameters on which this analysis is also based. In some cases, there were strong differences in the ranking of the individuals. There, the bibliometric parameters worked well to highlight the extremes—the “very top” and the “very bottom.” The arbitrariness of bibliometric parameters is particularly evident in the middle of the ranking (Fox and Seifert 2024). Judging science according to just one parameter does not seem fair. This must be considered in any analysis, consideration, or interpretation of science about science.

Furthermore, the data set is based exclusively on the “best scientist” ranking of *Research.com* and offers no guarantee for the completeness or accuracy of the data listed. The gender assignment was carried out manually and was a time-consuming process, which can still be subject to errors even after double-checking. One way of making such bibliometric (gender) analyses more transparent and simpler in the future would be to add information on gender to scientific databases. The same aspect should be considered for the other aspects analyzed, such as the exact subject area or the age. Transparency is the basis for reliable scientific analyses.

*The share of women and its background*

The women’s share in the analysis of the “best scientist” ranking in biology and biochemistry on *Research.com* is low at an average of 14.9%. Compared with previous studies and statistics by the UNESCO Institute for Statistics (2019), Zehetbauer et al. (2022), and Zöllner and Seifert (2023), this analysis results in an especially low female proportion. There are probably two main factors responsible for these differences. Firstly, the data set does not only deal with the latest research and includes historically significant scientists, some of whom have already passed away. One criticism of the h-Index is that it can also increase posthumously due to further citations of previously published works. For this reason, “old researchers” also remain in the list, because they have not (yet) been overtaken or they may even gain further places. The already mentioned vertical segregation is the second reason why the proportion of women is so low. The proportion of women decreases in the upper levels of academic structures, and elite research takes place in these ranks. The further up the hierarchy, the lower the share of women. The comparative studies mentioned above also show this pattern.

We defined different categories for the socio-economic factors leading to the different proportions of women in (elite) science: economic factors, rules, rights and laws, society and education, and country-specific, individual factors. These country-individual factors are summarized in Table 13.

In a recent study, the non-peer-reviewed science magazine *Biospektrum* was analyzed to also uncover gender ratios among the authors. Female pharmacologists in particular make little use of the opportunity to publish in the magazine, although it represents a minor obstacle compared to a publication in a peer-reviewed journal. These female scientists do not “lose” any “impact factor points” by not publishing in these journals, but they also do not gain any “social impact points”, which can open further career options (Zöllner and Seifert 2023). Regarding the analysis of *Research.com*, it can therefore be stated that background factors such as the lack of publication in non-peer-reviewed journals such as *Biospektrum* can also lead to a lower representation of women in science.

**Table 13** Further individual factors that could lead to the different female proportions in the analyzed countries

| Country (women’s proportion) | Individual socio-economic factors |
| --- | --- |
| Italy (31.2%) | Women were already allowed to attend universities in the 18th century. This is described as the “old roman spirit of freedom” (Cieslak-Golonka and Morten 2000). |
| Brazil (25.4%) | Brazil was a destination country for refugees of the Nazi regime, also important female researchers fled to Brazil (Plonski and Seidel 2001). |
| Poland (22.7%) | After 1945 and the establishment of the communist system, “white collar” laboratory jobs became appealing to women and their particiption rose. Economic crisis in 1980 and after 1990 lead to emigration of male scientists due to better working conditions elsewhere. The resulting vacuum of available positions in science was filled by women (Siemienska 2000). |
| South Africa (20%) | Dual problem: gender and race (Apartheid, history of institutional racism) (Mabandla, 2001). |
| Israel (18.2%) | The Jewish Law Halacha provides for a conservative role for women and clearly differentiates between gender roles in society. Young women also have experiences of the Israeli-Palestinian conflict (including in military training) that leave them with a mindset characterized by gender inequality (Cohen-Almagor and Maroshek-Klarman 2022). |
| Sweden (16.7%) | Carl Tham, former Swedish Minister of Education, created the 32 THAM-professorships for women in 1995. The idea that professorships are no longer limited but awarded according to status. Similar double strategy as Norway (Bladh 2001). |
| Belgium (15.2%) | The social situation is comparatively good for women and there are good childcare options (Pierrard 2005). |
| Russia (14.8%) | Gender equality was formally achieved after 1917 (Valkova 2008). Nevertheless, female scientists struggle with male-dominated structures and sexism (Pushkareva 2011). |
| Austria (14.5%) | The 2015 Amendment to the Universities Act stipulates a women's quota of at least 50% (Schaller-Steidl 2015). In 2005, the Ministry of Education and Research introduced the Excellentia programme, in which bonuses were paid for the appointment of female professors (Holzinger and Hafellner 2015). |
| Norway (14.3%) | Double strategy consisting of gender mainstreaming and special commitment (Søyland et al. 2001). |
| China (13%) | Growing enrolment of female students: mindset of universities has changed from elitism to massification. This has led to an increase in female participation in education (Lingyu et al. 2022). |
| Germany (11.9%) | Restricted access for women during the Third Reich with a decline in the women’s quota in education. Education reform in the 1960/70s led to mass education. Academic careers in Germany are described as a discontinuous, unpredictable process with unattractive working conditions (Fuchs et al., 2001). |
| USA (9.8%) | Double barriers exist for women of color in STEM (Ro et al., 2022). |
| Japan (4.1%) | Prime Minister Shinzu Abe’s ‘womenomics’ was intended to expand the female labour force to strengthen the economy. At its heart, Abe’s strategy was not aimed at breaking patriarchal structures but rather at improving Japan’s international standing and coping with demographic pressure (Arifin 2021). |
| Egypt (0%) | Underrepresentation of women in academia is a result of cultural barriers and institutional limitations (Mousa 2023). |

*Career paths of elite scientists*

The *Research.com* database is updated on a regular basis. This allows different versions of the scientist rankings to be compared with each other to track individual careers. To illustrate this point, the global top 100 from the original data set (2022 data collection) was compared with the global top 100 from the current ranking (2025 data collection, *Research.com* updated the data in November 2023). It is important to note that the comparison with this data may not be valid for all bibliometric factors, as the D-Index has been used since then. Thus, significant changes in the primary parameters are possible. Some positions in the rankings could also shift because of the methodological change. Nevertheless, to provide an outlook for future studies, this is presented as an example.

There has been some movement within the top 100. Twenty-six researchers who were in the top 100 in 2022 have dropped out. In return, there was room for 25 new researchers (a duplicate was found in the *Research.com* ranking, so this figure includes one less researcher). Compared to 2022, the number of women in the current ranking has increased by one (from five to six women). There has been little change in the origin of researchers. The proportion of US researchers has increased slightly, while the number of researchers of Asian origin has decreased. There has been little change in the European countries. Figure 17 illustrates these dynamics.

**Fig. 17** Origin of *Research.com*’s global top 100 scientists in biology and biochemistry, 2022 vs. 2025

The differences in the ranks of the individual scientists were then determined graphically and mathematically. The results are shown in Fig. 18 and Table 14. For the purposes of the analysis, the researchers in both years were first divided into three groups: the top 25 of the cohort (group A), the bottom 25 of the top 100 (group D), and the “midfield” of the cohort (group C). Because some researchers no longer appear in the 2025 ranking or appear in the 2025 ranking for the first time, it is not possible to provide information on change for these researchers. For this reason, the first and last 25 positions appearing in both rankings were also analyzed in a second step (group B and group E). This shows that the lower group moved up the most places from 2022 to 2025, but most of the scientists in this group also “dropped.” There was little movement in the upper ranks. The opposite is true for the 2025 distribution. Here, the upper ranks tended to move up, while the lower ranks tended to move down.

**Table 14** Mean rank change in *Research.com*’s “best scientist” ranking in biology and biochemistry 2022 vs. 2025

| Group | 2022 | | | 2025 | | |
| --- | --- | --- | --- | --- | --- | --- |
|  | Mean rank change | *N* | “Dropped out” | Mean rank change | *N* | Ascended |
| A: Rank 1–25 | −1.045 | 22 | 3 | 3.182 | 22 | 3 |
| B: Top 25 | −2.6 | 25 | / | 2.2 | 25 | / |
| C: Rank 26–75 | −4.659 | 41 | 9 | −1.421 | 38 | 11 |
| D: Rank 76–100 | 12.727 | 11 | 14 | −6.429 | 14 | 11 |
| E: Lower 25 | 7.92 | 25 | / | −7.52 | 25 | / |

**Fig. 18** Rank change (y) of the top 100 “best scientists” on *Research.com* in biology and biochemistry (x) from 2022 to 2025. The scientists appearing in both rankings are aligned horizontally, sorted from high rank in the 2022 ranking (left) to lower rank (right). Blue bars indicate that the scientist moved up in the 2025 ranking; red bars indicate a drop in ranking. The scientists’ rank changes are shown above the bars

**Conclusions**

The present analysis offers an extraordinary insight into bibliometric analyses of the top researchers in biology and biochemistry in an international comparison. To answer the questions mentioned above:

*1. Are there country-specific differences in the proportion of women in elite science?*

Women are underrepresented in all countries, in some even more than in others. So yes, there are country-specific differences in the proportion of women in elite science. There are numerous reasons for this, as listed in Table 10. Fundamental changes in the socio-cultural background of a country require time, which includes not only political but also social changes. Strategies for overcoming the underrepresentation of women in scientific professions are manifold: reforming childcare or making it more accessible, establishing mentoring programs, eliminating stereotypes from an early age, and creating special positions for women. It would be interesting to conduct a comparative study in a few years’ time to find out to what extent women have climbed the ladder of elite science. This also allows to follow career paths of individual researchers. The analysis of the global top 100 of 2022 compared to 2025 shows an overall moderate dynamic in the career paths of elite researchers. The composition remains largely constant in terms of geographical origin and gender distribution; a minimal change in the number of female researchers and a decrease in the number of Asian researchers illustrate the slow structural changes with present structural bias in bibliometric scientist rankings. The top positions stay relatively stable. However, because of the introduction of the D-Index, the evaluation remains limited, so annual comparisons should be used for dynamic analyses in the future.

*2. How do pharmacologists rank among elite scientists?*

Elite pharmacology is a small specialty in the main category of biologists and biochemists. The analyzed cohort consists only of men. Elite pharmacologists worldwide are older than scientists in other disciplines, even significantly older than the elite oncologists. This shows the popularity of a scientific discipline that is linked to different “cultures” in science. Pharmacologists receive fewer citations than other scientists, suggesting that pharmacology is less popular than other fields. This is also shown by the evaluation from a previous study: German pharmacologists have a significantly lower h/P-Index than German biologists and biochemists in the *Research.com* rankings (Fox and Seifert 2024).

*3. How do women perform in bibliometric analyses?*

Women are underrepresented in elite science particularly, which, in comparison to “normal science,” is also due to factors such as vertical segregation. On the other hand, however, women in the top list do not perform worse than men. They do not reach the top h-Index, but they are still able to establish themselves, as the analysis of the lower half of the big cohorts shows. And they are even characterized by a significantly higher h/P-Index worldwide. Based on previously published studies, this analysis therefore supports a very important statement: women are underrepresented in elite research, also in the Nobel laureate group, but they achieve good scientific recognition within this group (Bünemann and Seifert 2024).

*4. How comparable and fair are bibliometric analyses? Is the h/P-Index contributing useful information in bibliometric comparisons?*

A new way of assessing scientific quality was introduced, the h/P-Index. It was developed to obtain an indication of how many publications by a scientist are included in the determination of the h-Index. If the h/P-Index is close to 1, then almost all their publications achieve such a high number of citations (*h* number of citations) that they contribute to the h-Index or even drive it up. The h/P-Index is significantly higher for women worldwide, meaning women need fewer publications to reach the same h-Index as men.

This analysis deals exclusively with elite science: only researchers with an h-Index of at least 40 are included in the data set. This is already an extraordinary scientific achievement. In this cohort are some researchers that have hardly any publications that are cited “too little” and are not included in the determination of their h-Index. This is “complaining at a high level”: in an analysis of the top researchers, there are also publications that do not quite reach the extraordinary level, as the already very high h-Index suggests, to “contribute” something. It can therefore be assumed and observed that the h/P-Index tends to be slightly lower in the top ranks of the data set.

As with other bibliometric analysis parameters, an important issue is the subject-specific differences in publication culture. A work published by the Alexander von Humboldt Foundation addresses publication behavior in different scientific disciplines (Alexander von Humboldt-Stiftung 2009). It deals with the role of abstract quantification of the scientific achievements of researchers in comparison with peer review from the community. It should be concluded that bibliometric analyses supplement and specify the judgment of peers without being able to replace it. Furthermore, there is no “super indicator” of scientific quality; detailed data analyses of various aspects should supplement the overall picture. The extent to which the standards of scientific performance change depending on which discipline is considered is also examined.

The h/P-Index was developed against this background as a bibliometric parameter that includes both publications and citations and sets them in a relation. By dividing the h-Index, which is defined by the number of papers h with more than or equal to h citations, by the number of total publications P, part of which include the value h, a relative indication is generated of how consistently “good” the scientific quality of the scientists examined is. By including several primary parameters, multiple aspects of science are considered: productivity in terms of total publications, citations as a measure of the esteem a scientist receives from the scientific community, and the h-Index as a prolonged measure of scientific achievements in the career of a researcher. Quantitative and qualitative aspects are combined. However, the above-mentioned limitations of bibliometric analyses apply to cross-disciplinary analyses. Nevertheless, the new parameter offers an evaluation approach that deals with the consistency of scientific work and can therefore also be used for further analyses.

The h-Index is significantly influenced by the number of citations, publications, and the researchers’ age. Gender and country of employment are not significant parameters for determining the h-Index of a scientist in the global cohort, but gender is in the French elite group. Limitations like structural bias resulting in a high male and US-American proportion and incomplete age data may limit the generalizability of the results.

**Limitations**

The evaluation of a bibliometric ranking is judgmental in nature. As explained above, the authors do not support the premise that top researchers can be sufficiently characterized by top bibliometric performance. However, since we are addressing this very performance, it *must* be categorized and evaluated. It is clear, however, that a top scientist is characterized by much more than a large number of citations to their publications.

The dataset is based on the scientist ranking on *Research.com*. They state that they rely on OpenAlex and CrossRef as sources. They ensure a secure methodological process by manually checking the raw bibliometric data. The tools and platforms used for the comparison are not mentioned by name. The ranking is now in its 3rd edition, with the first edition published in 2014. At the time of data collection for the present analysis, the 2nd version was valid, since November 21, 2023, the current version with the switch to the D-Index. Furthermore, the limit of h-Index or D-Index greater than or equal to 40 was chosen based on the statements of Jorge E. Hirsch, as this is how he defines outstanding science (Hirsch 2005). However, it should be noted that the full methodology and data sources are not disclosed by *Research.com*, so the interpretation of the rankings should be done cautiously.

There are important limitations in the composition of the cohort due to the (exclusive) use of *Research.com* as a source:

1. The analyzed discipline

The assignment of researchers to disciplines is unclear and not well defined, especially since disciplines are sometimes very broadly structured. We have already identified a similar problem in a previous analysis of the Laborjournal ranking (Fox and Seifert 2024). Here we found that the ranking of the best pharmacologists also included pharmacists, so that a direct comparison was being made between two different disciplines. This was not indicated in the ranking, so the reader is unable to classify the information presented. A clear definition of the inclusion criteria of a researcher in a specific discipline would make the evaluation of the data much more transparent. This problem should already be reduced by the use of the D-Index on *Research.com*. However, the question remains as to how fair a direct comparison between the large group of biologists and biochemists is, since it is already known that some disciplines are significantly more popular subjects than pharmacology.

1. “Outstanding science”

The results cannot be generalized to the scientific community. This limitation of the transferability of the results is related to the study design. Researchers belonging to the elite (as defined by J. E. Hirsch) were deliberately chosen to study this elite bibliometrically and sociologically. A similarly structured study, which deals with “average scientists”, i.e., those who do not belong to outstanding science according to Hirsch’s definition, is a second analysis to be carried out. Here, the question is where and how such scientists are selected, so that one can speak of a random cohort.

1. Bibliometric bias in the cohort

By relying on a database based on bibliometric parameters that are subject to various influences, the cohort may be biased. For example, it can be assumed that factors such as age, gender, and country of origin or employment are proven determinants of the likelihood that a researcher’s scientific work will be recognized and—measured by metric criteria—receive attention in the form of citations. Previous studies have shown that it is more difficult for female authors to have their research published in major journals (Jagsi et al. 2006), that non-English research is subject to an “audience bias” (van Leeuwen et al. 2001), and that older researchers tend to perform better than those who are more recently established in their scientific careers due to the accumulation of metric analysis factors. In addition to this age factor, there is also the Matthew effect described by Cole and Cole (1973). It states that researchers receive more recognition and resources for their further research due to earlier scientific successes, so they tend to be more likely to achieve further successes. Older men who conduct research in English are therefore more likely to have an advantage in cohorts such as those compiled on *Research.com*. This is evident if one looks at the composition of the analyzed global top 100.

**Future studies**

This analysis forms the basis for further bibliometric and publication analyses.

The selection of countries already provides a comprehensive overview of the international situation, but the data set can be expanded or specified in future research. Subanalyses in the relevant countries can also be used to gain subject-specific insights, as was the case for the global top 100. It will also be particularly important to analyze the situation of pharmacology compared to biology and biochemistry in other countries than in Germany (Fox and Seifert, 2024). To perform this research, the pertinent member lists of national pharmacological societies must be analyzed.

Future versions of the *Research.com* rankings can be used to make annual comparisons of the (top) positions and to compare the proportion of women over the years, as shown above in an exemplary but possibly skewed way. Individual career paths can thus be presented in a comprehensible way. The basis for this is reliable and replicable data in the various versions, which is only partly the case in the current comparison.

Further data on which scientific analyses can be based can come in the form of analyses of scientific prizes. For example, the analysis by Halling et al. (2025) of scientific prizes awarded by German medical societies shows the gender gap that still exists in science. The Fritz Külz Prize, which is awarded in German pharmacology, is presented in more detail. It is awarded less often to women, but in recent years, the proportion of women awardees has increased, in line with the general trend in science. Here, too, the authors analyzed career paths: the prize appears to be a reliable indicator of potential scientific excellence, as 82.25% of awardees hold a professorship within at least 16 years after receiving the prize. Thus, analyses of scientific awards also show scientific potential or scientific performance.

Another aspect that could be carried out is a precise analysis of the publications of (elite) researchers. This would allow the quality of the publications to be broken down. Similar to the analysis of Zehetbauer et al. (2022) of the authors of Naunyn Schmiedeberg’s Archives of Pharmacology, a distinction could be made between the different positions of the authors. By differentiating the positions of the authors, a “quality analysis” of productivity could be performed if it is measured in terms of publications.

Other bibliometric parameters that can only be determined with reliable biographical data are age-dependent factors, such as the age-adjusted h-Index as a quotient of the h-Index and (academic) age. In this way, researchers who have been at the top for a long time and benefit from the Matthew effect can be distinguished from young, up-and-coming researchers. Here, too, a different, more reliable data source must form the basis of the research.

The Scientist Impact Factor (Lippi and Mattiuzzi 2017) can also be examined in future studies. For the number of articles published in a year x, the number of citations received by these articles in the two subsequent years x + 1 and x + 2 is divided by the number of articles published in year x. This gives the average number of citations a researcher can expect or receive for their work in the two subsequent years. This allows researchers’ current work to be compared without, for example, previous highly successful “breakthroughs” by older researchers distorting the bibliometric comparisons.

Ideally, there should be a bibliometric database that provides reliable and transparent bibliometric and biographical data for all researchers. One could imagine a directory that provides researchers (not just the elite) with information on their name, age, date of birth, gender, place of origin, and research location/affiliation, and the time when their academic career began. The gender of a researcher could also be recorded beyond the binary system. This would include transgender and non-binary identities. Due to the lack of information in the analyzed ranking, only binary assignments were possible, so there is room for further improvement and expansion of gender-sensitive research in the future. The discipline(s) that can be assigned to a researcher should also be listed by definition. A researcher can verify this information for him/herself, so that the user of the database has visual control. A photograph is used to identify the researcher. Bibliometric data includes the usual primary parameters with source and date, i.e., h-Index, publications, and citations. Variations of the h-Index, such as the D-Index or the age-adjusted h-Index, can also be found. The publications of the researcher are listed below. All important information for possible publication analyses can be found here: journal, discipline, citations, co-authors, and authorship position. The significance/quality of the publication can be classified by assigning an impact factor. Finally, an authorship network can be established/displayed by presenting frequent collaborations. This database should provide independent and verified biographical and bibliometric information about the scientists. Various filter mechanisms can be used to display rankings (e.g., fields, universities, gender, ...). With a database like this, transparent analyses can be performed.

**Supplementary information**

**Acknowledgements**

We would like to thank the DGPT for providing the member list and supporting the work.

**Authors Contributions**

L.C.F. and R.S. contributed to the study conception and design. L.C.F. performed data collection. L.C.F. performed data analysis. L.C.F. wrote the first draft of the paper. L.C.F. and R.S. commented on the first draft and read and approved the final manuscript. The authors declare that all data were generated in-house and that no paper mill was used.

**Data availability**

All source data for this study are available upon reasonable request.

**Declarations**

**Ethics approval**

This research was conducted according to the guidelines of good scientific practice of the Hannover Medical School (https://www.mhh.de/en/research/good-scientific-practice) and officially registered as a doctoral thesis project for Louisa Christin Fox. The Hannover Medical School follows the guidelines of the German Research Foundation (Deutsche Forschungsgemeinschaft, DFG).

**Consent for publication**

Not applicable because publicly available information is used as a data source.

**Competing interests**

L.C.F has no competing interests to declare. R.S. is the Editor-in-Chief of Naunyn-Schmiedebergs Archives of Pharmacology and follows the rules pertinent to editors acting as authors (https://link.springer.com/journal/210/submission-guidelines#Instructions%20for%20Authors_Competing%20Interests).

**References**

Alexander von Humboldt-Stiftung (2009) Publikationsverhalten in unterschiedlichen wissenschaftlichen Disziplinen: Beiträge zur Beurteilung von Forschungsleistungen. Alexander von Humboldt-Stiftung, Bonn

Alonso A, Diz I, Lois M (2016) Is gender mainstreaming helping women scientists? Evidences from research policies in Spain. Investigaciones Feministas 7(2):273–291. https://doi.org/10.5209/infe.52963

Arifin M (2021) Injustice continuity: gender inequality in Japan in the 21st century. HUMANISMA : Journal of Gender Studies 5(2):1–10. https://doi.org/10.30983/humanisme.v5i1.4355

Bladh A (2001). Gender and research in Sweden. In L. Maxwell, K. Slavin, & K. Young (Eds.), Gender & Research: Brussels, 8-9 November 2001 (pp. 35–36). Office for Official Publications of the European Communities

Boshoff N (2005) The representation of women academics in higher education in South Africa: progress in the pipeline? South African Journal of Higher Education 19(2):359–377. https://doi.org/10.4314/sajhe.v19i2.25658

Bünemann S, Seifert R (2024) Bibliometric comparison of Nobel Prize laureates in physiology or medicine and chemistry. Naunyn Schmiedebergs Arch Pharmacol. Advance online publication. https://doi.org/10.1007/s00210-024-03081-z

Cieslak-Golonka M, Morten B (2000) The women scientists of Bologna eighteenth-century. American Scientist 88(1):68–73.

Cohen-Almagur R, Maroshek-Klarman U (2022) Gender discrimination in Israel. In: Carby-Hall J, Góral Z, Tyc A (ed) Discrimination and Employment Law, 1st edn. Routledge, London, pp 248–276

Cole JR, Cole S (1973) Social stratification in science. University of Chicago Press, Chicago

Dashun W, Barabási AL (2021) The science of science. Cambridge University Press, Cambridge

Fox LC, Seifert R (2024) Arbitrariness of bibliometric parameters: a case study on leading scientists in the German Society for Experimental and Clinical Pharmacology and Toxicology (DGPT). Naunyn-Schmiedeberg's Arch Pharmacol 397(11):8925-8942. https://doi.org/10.1007/s00210-024-03195-4

Fuchs S, von Stebut J, Allmendinger J (2001) Gender, science, and scientific organizations in Germany. Minerva 39:175–201. https://doi.org/10.1023/A:1010380510013

Ghasemi A, Zahediasl S (2012) Normality tests for statistical analysis: a guide for non-statisticians. Int J Endocrinol Metab 10(2):486-489. https://doi.org/10.5812/ijem.3505

Grigat F (2018) Die USA sind weltweit das wichtigste Ziel für Wissenschaftler. Forschung & Lehre. https://www.forschung-und-lehre.de/politik/die-usa-sind-weltweit-das-wichtigste-ziel-fuer-wissenschaftler-829/. Accessed 8 February 2024

GraphPad Software (n.d.) Statistics guide: choosing a normality test. GraphPad Software. https://www.graphpad.com/guides/prism/latest/statistics/stat_choosing_a_normality_test.htm. Accessed 12 May 2025

Halling T, Mambrey V, Steinert JM, Seifert R, Dreher A, Marazia C, Loerbroks A, Hansson N (2025) The Gender Award Gap in German medical societies 2000-2023: the Fritz-Külz-Award as an example. Naunyn Schmeidebergs Arch Pharmacol. https://doi.org/10.1007/s00210-025-03892-8

Hirsch JE (2005) An index to quantify an individual’s scientific research output. Proc Natl Acad Sci U S A 102(46):16569–16572. https://doi.org/10.1073/pnas.0507655102

Holzinger F, Hafellner S (2015) Austria. In: Dubois-Shaik F, Fusulier B (ed) Academic careers and gender inequality: leaky pipeline and interrelated phenomena in seven European countries. University of Trento, Trento, pp 162-176

Ioannidis JP, Maniadis Z (2024) Quantitative research assessment: using metrics against gamed metrics. Intern Emerg Med 19:39–47. https://doi.org/10.1007/s11739-023-03447-w

Jagsi R, Guancial EA, Worobey CC, Henault LE; Chang Y, Starr R, Tarbell NJ, Hylek EM (2006) The “gender gap” in authorship of academic medical literature – a 35-year perspective. N Engl J Med 355(3):281-287. https://doi.org/10.1056/NEJMsa053910

Lingyu L, Wenquin S, Chao L (2022) The rise of women in STEM higher education in China: achievements and challenges. In: Ro HK, Fernandez F, Ramon EJ (ed) Gender Equity in STEM in Higher Education: International Perspectives on Policy, Institutional Culture, and Individual Choice, Routledge, pp 27–44)

Lippi G, Mattiuzzi C (2017) Scientist impact factor (SIF): a new metric for improving scientists’ evaluation? Ann Transl Med 5(15):303. https://doi.org/10.21037/atm.2017.06.24

Luukkonen-Gronow T (1987) University career opportunities for women in Finland in the 1980s. Acta Sociologica 30(2):193–206.

Mabandla B (2001) Gender and research in South Africa. In: Maxwell L, Slavin K, Young K (ed) Gender & Research: Brussels, 8-9 November 2001. Office for Publications of the European Communities, Luxembourg, pp 37–40

Mousa M (2023) From intersectionality to substantive representation: determinants of the representation of women faculty in academic contexts: voices of Egyptian academic leaders. International Journal of Public Administration 46(2):97–106. https://doi.org/10.1080/01900692.2021.1990317

Pierrard V (2005) Women in physics in Belgium. AIP Conference Proceedings 795:95–96. https://doi.org/10.1063/1.2128280

Plonski GA, Saidel RG (2001) Gender, science and technology in Brazil. Minerva 39:217–238.

Pushkareva N (2011) “Women scientists resemble guinea pigs…” Anecdotes about women-scientists in Soviet and Post-Soviet Russia. Gender forum 33:52–63.

Schaller-Steidl R (2015) From gender equality to diversity: current developments in Austrian higher education policy. In Wroblewski A (ed) Mentoring – an instrument to promote equality in science and research: status quo, new developments, and challenges. Institut für Höhere Studien, Wien, pp 28–31

Siemienska R (2000) Women in academe in Poland: winners among losers. Higher Education in Europe 25(2):163–172. https://doi.org/10.1080/713669262

Søyland A, Skarsbø AM, Amble N, Christensen L, Ølnes A (2000) Strategies for achieving gender equality in higher education and research in Norway. Higher Education in Europe 25(2):147–153. https://doi.org/10.1080/713669250

UNESCO Institute for Statistics (2019) Women in science. UNESCO Institute for Statistics. https://uis.unesco.org/sites/default/files/documents/fs55-women-in-science-2019-en.pdf. Accessed 7 February 2024

Valkova O (2008) The Conquest of Science: Women and Science in Russia, 1860-1940. Osiris 23(1):136–165. https://doi.org/10.1086/591872

van Leeuwen TN, Moed HF, Tijssen RJW, Visser MS, van Raan AFJ (2001) Language biases in the coverage of the Science Citation Index and its consequences for international comparisons of national research performance. Scientometrics 51(1):335-346. https://doi.org/10.1023/A:1010549719484

Van Noorden R, Maher B, Nuzzo R (2014) The top 100 papers. Nature 514(7524):550–553. https://doi.org/10.1038/514550a

VTC-Nachrichten (2023). Warum “halten“ Juden, die 0,2% der Weltbevölkerung ausmachen, die meisten Nobelpreise der Welt? Vietnamnet. https://www.vietnam.vn/de/chiem-02-dan-so-tai-sao-nguoi-do-thai-am-nhieu-giai-nobel-nhat-the-gioi/. Accessed 25 February 2024

Zehetbauer R, von Haugwitz F, Seifert R (2022) Gender-specific analysis of the authors and the editorial board of Naunyn-Schmiedeberg’s archives of Pharmacology from 2000 to 2020. Naunyn Schmiedebergs Arch Pharmacol 395(1):39–50. https://doi.org/10.1007/s00210-021-02166-3

Zöllner H, Seifert R (2023) How do German pharmacologists publish in the non-peer-reviewed science magazine Biospektrum? Naunyn Schmiedebergs Arch Pharmacol 397(3):1889–1900. https://doi.org/10.1007/s00210-023-02740-x
